# Improving service uptake and quality of care of integrated maternal health services: the Kenya kwale district improvement collaborative

**DOI:** 10.1186/1472-6963-14-416

**Published:** 2014-09-21

**Authors:** Michael K Mwaniki, Sonali Vaid, Isaac Mwamuye Chome, Dorcas Amolo, Youssef Tawfik, Kwale Improvement Coaches

**Affiliations:** University Research Co., LLC (URC), 7200 Wisconsin Avenue, Ste. 600, Bethesda, 20814 MD USA; Afya Research Africa, P.O. Box 20880, Nairobi, 00202 Kenya; Ministry of Medical Services and Ministry of Public Health and Sanitation, Government of Kenya, Afya House, Cathedral Road, P.O. Box: 30016-00100, Nairobi, Kenya

**Keywords:** Quality improvement, Antenatal care, Sub-Saharan Africa, Kenya

## Abstract

**Background:**

Health-related millennium development goals are off track in most of the countries in the sub-Saharan African region. Lack of access to, and low utilization of essential services and high-impact interventions, together with poor quality of health services, may be partially responsible for this lack of progress. We explored whether improvement approaches can be applied to increase utilization of antenatal care (ANC), health facility deliveries, prevention of mother-to-child transmission services and adherence to ANC standards of care in a rural district in Kenya. We targeted improvement of ANC services because ANC is a vital point of entry for most high-impact interventions targeting the pregnant mother.

**Methods:**

Healthcare workers in 21 public health facilities in Kwale District, Kenya formed improvement teams that met regularly to examine performance gaps in service delivery, identify root causes of such gaps, then develop and implement change ideas to address the gaps. Data were collected and entered into routine government registers by the teams on a daily basis. Data were abstracted from the government registers monthly to evaluate 20 indicators of care quality for improvement activities. For the purposes of this study, aggregate data for the district were collected from the District Health Management Office.

**Results:**

The number of pregnant mothers starting ANC within the first trimester and those completing at least four ANC checkups increased significantly (from 41 (8%) to 118 (24%) p=0.002 and from 186 (37%) to 316 (64%) p<0.001, respectively). The proportions of ANC visits in which provision of care adhered to the required standards increased from <40% to 80-100% within three to six months (X^2^ for trend 4.07, p<0.001). There was also a significant increase in the number of pregnant women delivering in health facilities each month from 164 (33%) to 259 (52%) (p=0.012).

**Conclusion:**

Improvement approaches can be applied in rural health care facilities in low-income settings to increase utilization of services and adherence to standards of care. Using the quality improvement methodology to target integrated health services is feasible. Longer follow-up periods are needed to gather more evidence on the sustainability of quality improvement initiatives in low-income countries.

**Electronic supplementary material:**

The online version of this article (doi:10.1186/1472-6963-14-416) contains supplementary material, which is available to authorized users.

## Background

A significant proportion of the global burden of preventable morbidity and mortality occurs in low-income countries with some of the highest rates being in the sub-Saharan African region [1]. Most nationally led efforts towards addressing the health related millennium development goals have rightfully focused on providing the necessary inputs (management functions, training and recruiting personnel, building more facilities, and providing more equipment etc.) [2, 3]. Donor-driven efforts have also been heavily skewed towards provisions of necessary inputs [4]. Despite this, most countries especially in sub-Saharan Africa have only made modest gains towards achieving the aspirations of the health-related millennium development goals [5, 6].

Over the last few decades, there has also been a growing drive towards examining the processes of care and improving these processes (quality improvement) in addition to providing inputs with an aim of improving health outcomes [7–9]. Considerable experiences and results exist on applying quality improvement in resource-rich country settings [10–14]. Adaptation and applications of similar methodologies in middle- and low-income countries have also demonstrated reasonable success [8]. However, there are several shortcomings of most of the documented evidence on application of quality improvement to strengthen the health sector in low-income country settings. First, many published studies have mainly provided data on the application of quality improvement to single technical areas such as scaling up active management of third stage of labor, as opposed to integrated health care services [9]. Second, these studies have mainly concentrated on adherence to standards and guidelines [8, 9]. It is worth noting that a majority of the inhabitants in low-income countries especially in sub-Saharan Africa may not reach the health care facility and seek alternative health care from traditional healers/practitioners and traditional birth attendants [15–17]. Therefore concentrating solely on adherence to clinical standards at points of care without deliberate attention to increasing utilization of critical services such as antenatal care (ANC), skilled delivery, prevention of mother-to-child transmission of HIV/AIDS (PMTCT), and early newborn care, among others, may not have a significant impact on population health outcomes in rural resource-poor settings.

Kenya is a low-income country in sub-Saharan Africa. The country has some of the poorest health indicators and overall is not on track towards achieving most of the health related millennium development goals [18]. The health delivery system in the country is organized in a hierarchical pattern with six distinct levels [19]. The community health activities are categorized as level one, while the national referral hospitals are level six. Most health facilities in the country are level 2 (dispensaries) and level 3 (health centers) [19]. These facilities are managed by approximately two to four nurses only. Each district may have many of such small facilities with one district hospital (level 4). A similar arrangement is seen in other sub-Saharan African countries [20, 21].

Although the country’s health indicators are generally poor, regional disparities exist. Rural districts that account for 60% of the population tend to perform worse than urban centers [18]. Given this scenario, we specifically sought to examine the application of quality improvement to increase utilization of integrated health services (ANC, PMTCT, and skilled delivery) and improve adherence to clinical standards and guidelines in an entire rural district over a 20-month period from January 2011 to August 2012. For this intervention, the Ministry of Health-Kenya identified a rural district (Kwale, district,) that was performing below the national average in most of the health indicators.

## Methods

### Context

Kwale district comprises two administrative divisions (Matuga and Kubo) with a combined total population of close to 160,000 [22]. The district is one of the poorest in the country with close to 50% of its inhabitants classified in the absolute poverty category [22]. Compared to the national average, the district has some of the poorest health indicators. Estimates put the infant mortality rate at about 90 per 1,000 live births compared to 77 per 1,000 live births nationally [22]. The child mortality rate is as high as 100–120 per 1,000 live births whilst the national rate is 74 per 1,000 live births [22]. Maternal mortality ratio is also high at 590-700/100,000 live births compared to the national average of 488 maternal deaths per 100,000 live births [22]. Most of the pregnant women start their ANC care visits late, and hence more than two thirds of all women never complete their scheduled antenatal care visits. Importantly, a sizeable proportion of those attending ANC clinics do not receive essential services such as having their ANC profiles done [23]. Furthermore, only one third of the estimated 6,000 annual deliveries in the district are assisted by a skilled health worker [22, 23]. Malaria contributes close to 40% of outpatient morbidity overall [22]. HIV prevalence is estimated at about 4% [24]. The majority of the inhabitants have difficulty in accessing health care facilities due to long distances [22]. Out-of-pocket payment for health care services is sometimes a financial barrier [22].

### Study design

In this study we set out to examine whether quality improvement approaches can be applied to increase utilization of integrated health services (ANC, PMTCT, and skilled delivery) and improve adherence to clinical standards and guidelines in a rural district. Furthermore we wanted to determine if this can be achieved within the confines of the routine supportive supervision set up. To achieve this, we undertook a pre- and post-implementation evaluation of the impact of quality improvement activities on improving the above services.

### Sample selection-the improvement collaborative

An improvement collaborative consists of a group of health workers drawn from different health facilities that work on the same set of indicators and meet regularly (usually every 3–6 months) to share working ideas [25]. This allows rapid diffusion of such ideas and their replication by other facilities in the collaborative. Through this approach, large scale district-wide improvement can be realized faster. All Ministry of Health facilities in Kwale District were included in this activity: one government-run hospital, three health centers and 17 dispensaries. These 21 facilities constituted the Kwale improvement collaborative.

### Implementation process

The district health office is managed by the District Health Management Team (DHMT) led by the District Medical Officer for Health (DMOH). The DHMT oversees health resources and services in the district and has overall responsibility to improve the health status of the community. For sustainability, all activities under this project were carried out by the Kwale DHMT with technical support from the United States Agency for International Development (USAID) Health Care Improvement Project (HCI).

### Training of the DHMT on application of quality improvement approaches in healthcare

HCI provided one week training on quality improvement to the DHMT. This training involved core aspects of quality improvement such as:- applying system thinking as healthcare managers, using data to identify quality gaps, application of various quality improvement tools (process maps, the Ishikawa diagram, Pareto charts among others), developing an improvement plan, how to come up with change ideas and put them through the Plan-Do-Study-Act cycle, measuring improvement, and how to set up and mentor/coach improvement teams [25].

The DHMT members then worked with each of the 21 facilities in the district to assist them in forming a 7–12 member quality improvement team composed of facility health-care personnel, community health-care volunteers, and community representatives from the given facility’s catchment area. These 21 facility based improvement teams were to work on the same indicators and hence form a ‘collaborative’. The DMOH through a consultative process finally assigned each of the trained DHMT members two or three improvement teams to mentor/coach on quality improvement as part of their regular supportive supervision.

### Selection of Indicators to monitor improvement progress

As part of the implementation plan, the DHMT selected a set of 20 indicators to be used to monitor progress (Additional file 1). These were primary indicators that the district is required to routinely report on for ANC and related programs in Kenya.

### Operations of the facility level improvement teams and development of change ideas

The facility improvement teams were continuously mentored/coached by the DHMT members on how to rigorously examine the process of service delivery, identify root causes of any problems, and finally develop and implement change ideas addressing the problem. The team members met at least once fortnightly to review progress and plotted their data monthly to monitor the impact of any changes that had been implemented. Importantly, each team documented every single change idea they were developing and testing in their improvement files. A quality improvement advisor from HCI provided ongoing support and guidance to the DHMT in carrying out activities for the duration of the project. HCI also provided financial support to cater for sharing forums and met the minimal field transport costs for the DHMT to enable them visit and coach their respective facilities at least once a month.

Sharing forums were organized every 3–4 months in line with the requirements of an improvement collaborative [8, 25]. During these sessions, representatives from all 21 teams met and exchanged their successes and challenges in improving care. These forums enabled diffusion of emerging working ideas across sites. At the end of the project all change ideas tested by teams were reviewed. The teams further ranked each of the change ideas to determine their feasibility and identify ideas that can be recommended to other districts in similar settings. Ranking was based on four parameters:- i) Number of sites that implemented the specific idea. Therefore an idea that was implemented and shown to be working by more sites scored higher. ii) Simplicity/how easy it was for the team to implement the idea. Iii) Scalability, how easily the idea could be replicated in other similar settings. iv) Relative importance, its contribution to the results achieved. Each parameter had 5 as the highest score and 1 as the lowest; therefore the maximum score an idea could get was 20 and a minimum 4. Finally a detailed guide of how each change idea was implemented was prepared (Additional file 2). This guide will be used in the dissemination of these change ideas to other districts keen on improving their services and further inform the national Ministry of Health on how to implement quality improvement approaches in rural settings in Kenya.

### Changes implemented

A large number of change ideas were developed and implemented by health facilities teams for the five key focus areas of intervention: ANC coverage, ANC quality of care, health facility deliveries, PMTCT, and community linkages (Additional file 2). These ideas were shared among all teams, and multiple teams tried the same ideas. Some of the successful change ideas implemented by teams and rated highly using the criteria described above are summarized in Table 1.

**Table 1:** **Highly ranked change ideas recommended by the collaborative for implementation in similar settings**

Focus area	Change idea implemented
Utilization of ANC and skilled delivery services and health facility-community linkages.	i. The facility staffs engaged traditional birth attendants (TBAs) in dialogue and redefined their roles to those of birth companions who would help trace women for missed appointments and accompany women to the health facility for skilled deliveries.
	ii. All facilities introduced the routine practice of asking all women of reproductive age coming to the health facility for other health services (general outpatient visits, immunization visits) or their last menstrual period to identify pregnant women.
	iii. Health workers gave their phone numbers to TBAs and community health workers (CHWs) to ease communication anytime they were bringing clients to the health facility. A dedicated health facility phone was also used to follow up on ANC clients and remind them of their appointments.
	iv. After discussion with the community, mothers who could not afford to pay for ANC laboratory tests at once were allowed to pay in installments.
Quality of care at the health facility	i. All facilities drastically reduced client waiting time by process mapping their services, eliminating unnecessary steps, and providing integrated ANC care in one room.
	ii. Some health facilities took the initiative to submit proposals for financial support from local donors, such as residents’ associations, local banks and traders’ groups, to overcome resource constraints and also lobbied local politicians and other stakeholders for the provision of items such as microscopes to equip laboratories. Three new laboratories were started due to these efforts.
	iii. Due to the unreliable government supply of iron and folate, facilities reallocated their funds and purchased these items locally since they were cheap and critical components of ANC.
	iv. Almost all healthcare staff in the district are trained in providing services. However Antiretroviral drugs (ARVs) were only available at the district’s central pharmacy. The health management team reviewed this protocol/policy such that that all health facilities were allowed to stock a limited number of ARVs for better implementation of PMTCT services across the entire district.
	v. Clean water and cups were provided in the ANC room so that the ANC clients could take sulphadoxine-pyrimethamine for malaria prophylaxis under direct observation of the health worker. This way they did not have to queue again at the pharmacy for these drugs.
	vi. Staff in all the facilities initiated regular structured dialogues with various community groups (women groups, traditional birth attendants and opinion leaders) and also used general community meetings to get open criticism on services and their attitudes.
	vii. Privacy during delivery was enhanced by designating rooms for delivery, having curtains in place, and restricting entrance to the delivery rooms.

Certain change ideas were more challenging to implement. For example attempts were made to integrate antenatal care services into outreach services for vaccination but this idea faced logistical challenges and was not done by most facilities. Although some facilities improved 24-hour coverage by rearranging staff duty schedules and moving the staff members to the staff quarters on facility premises, this was not feasible in all facilities due to shortage of personnel, security concerns and lack of staff quarters. Importantly, to tackle financial barriers, some facilities tested the feasibility of staggering any required payments over the entire pregnancy period rather than asking the mothers to pay the entire amount at once. Overall the teams developed and tested over fifty change ideas. A full description of each of the change idea is provided (Additional file 2).

### Ethical considerations

Given that this project was embedded within the Government of Kenya, Ministry of Health’s routine improvement and supportive supervision strategy, and that regularly collected, anonymized secondary data were used for the analysis with no patient contact; the study was exempt from Institutional Review Board submission according to the Office of Human Research Protection (OHRP) guidelines [26].

### Data collection

Twenty indicators targeting integrated reproductive health services were selected by the DHMT to be used by the improvement teams in monitoring their facilities’ progress in improving access and quality of care (Additional file 1). Data used to calculate the 20 indicators were collected and entered into routine government registers by the quality improvement teams on a daily basis. The data were abstracted monthly by the health facility staff from the government registers for quality improvement activities. For the purposes of this study, aggregated, anonymous data for the district were collected from the records of the District Health Management Office.

### Statistical analysis

Raw data on the 20 indicators in the five technical focus areas were collected and entered into routine government registers by the quality improvement teams daily. Aggregate district data were obtained from the district health information office. The aggregate data were then entered into excel spreadsheets for preliminary manipulation then transferred into STATA 11.0 (Stata Corp, College Station, TX) for further analysis. We compared proportions using the x^2^ test or Fisher exact test where appropriate and further explored for trends using the x^2^ test for trend. Ministry of Health estimates for denominators for each of the health facilities’ catchment area were used in calculating coverage.

## Results

### Antenatal care coverage

**Figure 1:**
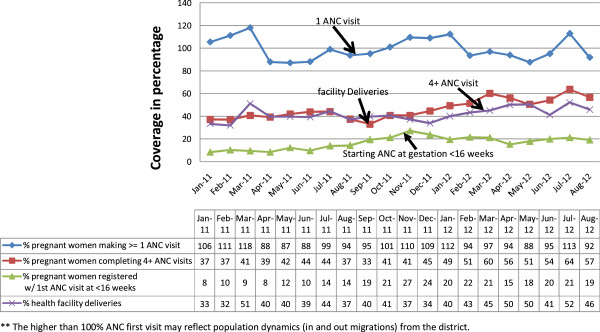
**Antenatal care (ANC) and skilled delivery coverage.**

There was marked increase in ANC coverage (Figure 1). The percentage of pregnant women attending at least one ANC visit remained high (90%-100%) throughout the implementation period. Importantly, the percentage of pregnant women accessing ANC within the first trimester (<16 weeks of gestation) significantly increased from 41 (8%) to 118 (24%) (x^2^ for trend 3.49, p 0.002). Similarly, there was a significant increase among those completing four ANC visits, from 186 (37%) to 316 (64%) (X^2^ for trend 3.64, p < 0.001).

### Quality of antenatal care

**Figure 2:**
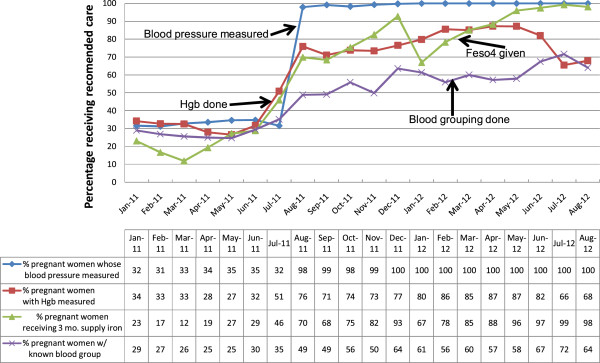
**Pregnant women receiving recommended care during ANC visits.**

Eight parameters were tracked to monitor the quality of care given to pregnant women attending ANC visits. This included: hemoglobin measurement, blood grouping, blood pressure measurement, provision of three months’ supply of iron and folate, administration of at least two doses of tetanus toxoid vaccination, two doses of prophylaxis against malaria, supply of long-lasting insecticide-treated nets, and counseling (on danger signs of pregnancy, birth preparedness, PMTCTand the need for postnatal family planning). The proportions of ANC visits per month in which provision of care adhered to the required standards for all the above parameters increased from 454 (<40%) to 1366 (80-100%) within three to six months (X^2^ for trend 4.07, p < 0.001). This improvement was sustained throughout the 20 months for which the data were collected. The aggregate results are summarized in Figure 2.

### Skilled deliveries

The percentage of deliveries assisted by a skilled health worker nearly doubled from 164 (33%) to 259 (52%) (X^2^ for trend 2.51, p = 0.012) (Figure 1). Increase in the percentage of pregnant women preferring to deliver at the health facilities closely mirrored those completing at least four or more ANC visits.

### Community referrals

**Figure 3:**
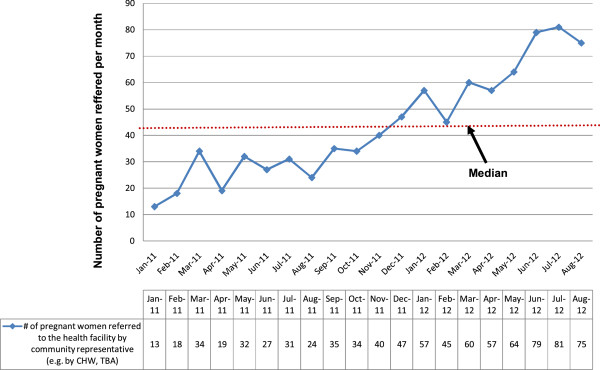
**Number of pregnant women referred to health facilities for ANC or skilled deliveries by community representatives.**

The number of pregnant women actively referred from the community to health facilities for ANC services and skilled deliveries significantly increased from 13 per month to 75 per month (X^2^ for trend 4.12, p < 0.001) (Figure 3).

## Discussion

In this improvement effort, simple interventions developed and tested by frontline health workers were effective in increasing access to health services and also in improving adherence to standards of care for maternal health. Notably, these interventions were implemented both at the facilities and catchment communities creating a unique continuum of care in a rural district. These improvement efforts were led by DHMT members who acted as coaches/mentors of the improvement teams. This approach thus strengthened their leadership skills enabling them to optimize the utilization of the limited available resources to produce better outcomes by improving the processes of care.

Physical and financial access to care may be an important contributor to health outcomes [27–30]. In most low-income countries lack of access to skilled care may be a significant factor behind high morbidity and mortality [30, 31]. Therefore our approach in Kwale combined strategies for improving adherence to standards at points of care with measures to work with the community and increase access. Simple strategies such as allowing pregnant women to make any required payments in installments were seen to be highly feasible in such rural remote settings and could contribute significantly to improving access to care. Furthermore involving the community resource persons in “marketing” the facility as a preferred destination for health services through regular structured dialogues with the various community groups can also rapidly lead to increased utilization of high impact health services and interventions. Such dialogues were used to discuss any complaints and suggestions from the community on how best to improve the quality of health services in Kwale district. Through the dialogues facilities together with the community would develop, plan and test any agreed changes.

Apart from access, many more barriers that discourage pregnant women from utilizing rural health facilities were recognized [32]. Some of them include complaints around health care staff attitudes, lack of privacy during delivery, denying trusted companions such as mothers-in-law access to the delivery rooms during the period of labor and delivery, lack of facilities for the mother to clean herself after delivery, and even something to eat after delivery. Through the structured community dialogues the facility improvement teams were able to get feedback on such critical issues including reports of abuse during delivery, and take corrective action. Furthermore simple ideas such as allowing trusted companions into delivery room, warming water for mothers to clean up after delivery and providing some hot refreshment especially porridge were easily accommodated.

Waiting time and delays at actual point of service delivery is an impediment to providing quality care [32]. Through process mapping of their services, improvement teams were able to identify and eliminate unnecessary steps and further integrate related services. Overall the process mapping tool may not only lead to reduced client waiting time, but in such rural health facilities that are struggling with staff constraints, it may also reduce overall staff work load.

Most facilities in rural districts in low income countries are small dispensaries staffed by one to four nurses only [19–21]. Despite these staff shortages, they are still expected to offer a wide spectrum of integrated health care services including ANC, PMTCT, skilled deliveries, immunization, counseling, public health promotion and preventive services, and also provide general clinical care. Our approach of strengthening the integration of PMTCT with ANC and skilled deliveries is therefore consistent with the principle of offering comprehensive integrated services even in primary care settings [33, 34]. Therefore the Kwale improvement approach which holistically targeted a reasonable range of integrated health services that are routinely offered in small rural health facilities in resource constrained settings, provided further evidence that this is possible.

### Limitations

A major drawback of our report is that we had a limited period of implementation and follow-up. However, a recent review of quality improvement initiatives showed that it is possible to improve care with specific reference to adherence to standards within a short period of time (9 months), and also demonstrated that such gains can be sustained [8]. However, larger projects with longer follow-up periods especially at the end of any direct funding or technical assistance are needed to gather more evidence on the sustainability of quality improvement initiatives in low-income countries. It is also worth noting that this improvement collaborative focused on improving the quality of care from the point of increasing utilization, and ensuring that pregnant women receive requisite services when they attend ANC clinics and other maternal health related services. Such follow up studies should also be structured to document effect on long-term outcomes such as perinatal mortality among others. In addition, they should explore the inclusion of appropriate control districts/sites in order to provide more robust findings. Finally, we did not have true denominators of the number of pregnant women and relied on Government of Kenya Ministry of Health estimates for the catchment population for each health facility. This may have led to an under or overestimation of improved coverage.

## Conclusion

Our results suggest that improving health care is possible in very small rural health facilities in low-income settings without provision of external resources. Our findings further demonstrate that quality improvement principles and approaches can be applied to target increasing access and improving adherence to standards at points of care. Overall, our results provide further evidence for the value of quality improvement approaches in health care service delivery in low-income countries as a core part of the repertoire of strategies aimed at accelerating efforts towards achievement of the health related millennium development goals. Longer follow up studies are however needed to elucidate how such strategies can be sustained and their impact on core outcomes such as maternal and neonatal mortality among others.

## Electronic supplementary material

Additional file 1: **Improvement Teams Indicators Tracking Template.** (DOC 96 KB)

Additional file 2: **Change Package for Improving the Quality of Antenatal Care Services and Skilled Deliveries in Kwale, Kenya.** (DOCX 397 KB)

## References

[1] Murray CJL, Vos T, Lozano R, Naghavi M, Flaxman AD, Michaud C, Ezzati M, Shibuya K, Salomon JA, Abdalla S, Aboyans V, Abraham J, Ackerman I, Aggarwal R, Ahn SY, Ali MK, Alvarado M, Anderson HR, Anderson LM, Andrews KG, Atkinson C, Baddour LM, Bahalim AN, Barker-Collo S, Barrero LH, Bartels DH, Basáñez M-G, Baxter A, Bell ML (2012). Disability-adjusted life years (DALYs) for 291
diseases and injuries in 21 regions, 1990–2010: a systematic analysis for the
global burden of disease study 2010. Lancet.

[2] Lu C, Schneider MT, Gubbins P, Leach-Kemon K, Jamison D, Murray CJL (2010). Public financing of health in developing countries: a
cross-national systematic analysis. Lancet.

[3] *“Microsoft Word - KENYA HEALTH POLICY Final Draft - kenya health policy.pdf”. [Online]*. 2013. Available: http://countryoffice.unfpa.org/kenya/drive/FinalKenyaHealthPolicyBook.pdf. [Accessed: 27-Mar-2013]

[4] Sambo LG, Kirigia JM, Ki-Zerbo G (2011). “Health financing in Africa: overview of a dialogue
among high level policy makers”. BMC Proc.

[5] Lozano R, Wang H, Foreman KJ, Rajaratnam JK, Naghavi M, Marcus JR, Dwyer-Lindgren L, Lofgren KT, Phillips D, Atkinson C, Lopez AD, Murray CJL (2011). Progress towards millennium development goals 4 and 5
on maternal and child mortality: an updated systematic analysis. Lancet.

[6] Hogan MC, Foreman KJ, Naghavi M, Ahn SY, Wang M, Makela SM, Lopez AD, Lozano R, Murray CJL (2010). Maternal mortality for 181 countries, 1980–2008: a
systematic analysis of progress towards millennium development goal
5. Lancet.

[7] Berwanger O, Guimarães HP, Laranjeira LN, Cavalcanti AB, Kodama AA, Zazula AD, Santucci EV, Victor E, Tenuta M, Carvalho V, Mira VL, Pieper KS, Weber B, Mota LH, Peterson ED, Lopes RD (2012). Effect of a multifaceted intervention on use of
evidence-based therapies in patients with acute coronary syndromes in Brazil:
the BRIDGE-ACS randomized trial. JAMA.

[8] Franco LM, Marquez L (2011). Effectiveness of collaborative improvement: evidence
from 27 applications in 12 less-developed and middle-income
countries. BMJ Qual Saf.

[9] Hermida J, Salas B, Sloan NL (2012). Sustainable scale-up of active management of the third
stage of labor for prevention of postpartum hemorrhage in Ecuador. Int J Gynaecol Obstet.

[10] Mangino JE, Peyrani P, Ford KD, Kett DH, Zervos MJ, Welch VL, Scerpella EG, Ramirez JA (2011). Development and implementation of a performance
improvement project in adult intensive care units: overview of the Improving
Medicine Through Pathway Assessment of Critical Therapy in Hospital-Acquired
Pneumonia (IMPACT-HAP) study”. Crit Care.

[11] Brandt KL, Booker JM, McGrath J (2013). Clinical Quality Improvement for Identification and
Management of Overweight in Pediatric Primary Care Practices”. Clin Pediatr (Phila).

[12] Siegelman JRQW, Gress DA (2013). Radiology Stewardship and Quality Improvement: The
Process and Costs of Implementing a CT Radiation Dose Optimization Committee in
a Medium-Sized Community Hospital System. J Am Coll Radiol.

[13] Compoginis JM, Katz SG (2013). American college of surgeons national surgical quality
improvement program as a quality improvement tool: a single Institution’s
experience with vascular surgical site infections. Am Surg.

[14] Unbeck M, Sterner E, Elg M, Fossum B, Thor J, Pukk Härenstam K (2013). Design, application and impact of quality improvement
‘theme months’ in orthopaedic nursing: a mixed method case study on pressure
ulcer prevention. Int J Nurs Stud.

[15] Chukwuneke FN, Ezeonu CT, Onyire BN, Ezeonu PO (2012). Culture and biomedical care in Africa: the influence
of culture on biomedical care in a traditional African society, Nigeria, West
Africa”. Niger J Med.

[16] de-Graft Aikins A (2005). Healer shopping in Africa: new evidence from
rural–urban qualitative study of Ghanaian diabetes experiences”. BMJ.

[17] Nylander PP, Adekunle AO (1990). Antenatal care in developing countries. Baillieres Clin Obstet Gynaecol.

[18] *Kenya Demographic and Health Survey 2008–09 [FR229] - FR229.pdf”. [Online]*. 2013. Available: http://dhsprogram.com/pubs/pdf/FR229/FR229.pdf. [Accessed: 06-May-2013]

[19] **“Overview of the Health System in Kenya.”** 2013.http://dhsprogram.com/pibs/pdf/spa8/02chapter2.pdf

[20] Stephen M, Grace C, Rebecca P, Emmanuel M, Dereck C, Eric Van P, Greta S (2013). Tanzania Health System Assessment 2010 Report. Abt Associates
Inc.”.

[21] Gilbert K, Lisa F, Eddie K, Aneesa A, Parsa S, Ligia P, Lola D, Ahmed A, Shekwoduza B, Eno U, Sam U, USAID (2013). Nigeria Health System Assessment 2008. Abt Associates Inc”.

[22] *“KWALE_DISTRICT_STRATEGIC_PLAN.pdf”. [Online]*. 2013. Available: http://www.kecosce.org/downloads/KWALE_DISTRICT_STRATEGIC_PLAN.pdf. [Accessed: 29-Mar-2013]

[23] Brown CA, Sohani SB, Khan K, Lilford R, Mukhwana W (2008). Antenatal care and perinatal outcomes in Kwale
district, Kenya. BMC Pregnancy Childbirth.

[24] *“Kenya Aids Indicator Survey_report_2009.pdf”. [Online]*. 2013. Available: http://nascop.or.ke/library/3d/Official_KAIS_Report_20091.pdf. [Accessed: 29-Mar-2013]

[25] I. for H. Boston: Improvement, “No
Title,” (2013). The Breakthrough Series: IHI’s Collaborative Model for Achieving
Breakthrough Improvement, 2003. [Online].

[26] O. for H. R. P. (OHRP): **“Office for Human Research Protections (OHRP).** 10.1038/79605 10973291

[27] Kyei NNA, Campbell OMR, Gabrysch S (2012). The influence of distance and level of service
provision on antenatal care use in rural Zambia”. PLoS ONE.

[28] Gabrysch S, Simushi V, Campbell OMR (2011). Availability and distribution of, and geographic
access to emergency obstetric care in Zambia. Int J Gynaecol Obstet.

[29] Gething PW, Johnson FA, Frempong-Ainguah F, Nyarko P, Baschieri A, Aboagye P, Falkingham J, Matthews Z, Atkinson PM (2012). Geographical access to care at birth in Ghana: a
barrier to safe motherhood. BMC Public Health.

[30] Pearson L, Shoo R (2005). Availability and use of emergency obstetric services:
Kenya, Rwanda, Southern Sudan, and Uganda. Int J Gynaecol Obstet.

[31] Okwaraji YB, Edmond KM: **“Proximity to health services and child survival in low- and middle-income countries: a systematic review and meta-analysis”.** *BMJ Open* 2012.,**2**(4)**:** 10.1136/bmjopen-2012-001196 PMC3400076 22798257

[32] Thaddeus S, Maine D (1994). Too far to walk: maternal mortality in
context. Soc Sci Med.

[33] *GHI Principle Paper on Integration in the Health Sector - 195596.pdf”. [Online]*. 2013. Available: http://www.ghi.gov/principles/docs/principlePaperIntegration.pdf. [Accessed: 29-Mar-2013]

[34] *“Pan American Health Organization ‘Integrated Health Service Delivery Networks: Concepts, Policy Options and a Road Map for Implementation in the Americas’ Washington, D.C.: PAHO, © 2011”. [Online]*. 2013. Available: http://www2.paho.org/hq/dmdocuments/2011/PHC_IHSD-2011Serie4.pdf. [Accessed: 29-Mar-2013]

[CR35] The pre-publication history for this paper can be accessed here:http://www.biomedcentral.com/1472-6963/14/416/prepub

