# Supplementary material for: Improving service uptake and quality of care of integrated maternal health services: the Kenya kwale district improvement collaborative
Source: BMC Health Serv Res. 2014 Sep 21;14:416. doi: 10.1186/1472-6963-14-416 (PMC4179240; doi:10.1186/1472-6963-14-416)
Supplement: Supplementary file 1 — Additional file 1: Improvement Teams Indicators Tracking Template. (DOC 96 KB) [file 12913_2013_3504_MOESM1_ESM.doc]

**Additional file 1: Improvement Teams Indicators Tracking Template**

DISTRICT: __________________________________ FACILITY NAME: _________________________________ Year ________________

|  |  | Jan | Feb | Mar | Apr | May | June | July | Aug | Sept | Oct | Nov | Dec | Data Source |
| --- | --- | --- | --- | --- | --- | --- | --- | --- | --- | --- | --- | --- | --- | --- |
| **A** | **ANC Coverage** |  |  |  |  |  |  |  |  |  |  |  |  |  |
| *i* | *Estimated # pregnant women in the catchment area** |  |  |  |  |  |  |  |  |  |  |  |  |  |
| 1 | # attending ANC 1 visit per month  (= new ANC clients per month) |  |  |  |  |  |  |  |  |  |  |  |  |  |
| 2 | # completing at least ANC 4 visits per month |  |  |  |  |  |  |  |  |  |  |  |  |  |
| **B** | **Delivery** |  |  |  |  |  |  |  |  |  |  |  |  |  |
| *ii* | *# of expected deliveries in the catchment area per month** |  |  |  |  |  |  |  |  |  |  |  |  |  |
| 3 | # of facility deliveries per month |  |  |  |  |  |  |  |  |  |  |  |  |  |
| 4 | # referred for delivery elsewhere **(Include mothers who came and go back because of any reason e.g no staff etc)** |  |  |  |  |  |  |  |  |  |  |  |  |  |
| **C** | **Preventive Care** |  |  |  |  |  |  |  |  |  |  |  |  |  |
| *iii* | *Total # ANC visits at facility per month (= new + revisit)* |  |  |  |  |  |  |  |  |  |  |  |  |  |
| 5 | # with blood pressure reading documented per month |  |  |  |  |  |  |  |  |  |  |  |  |  |
| 6 | # with haemoglobin level documented per month |  |  |  |  |  |  |  |  |  |  |  |  |  |
| 7 | # receiving 3 months supply of iron supplements per month |  |  |  |  |  |  |  |  |  |  |  |  |  |
| 8 | # receiving 3 months supply of folate supplements per month |  |  |  |  |  |  |  |  |  |  |  |  |  |
| 9 | # with blood group (including Rh factor) documented per month |  |  |  |  |  |  |  |  |  |  |  |  |  |
| 10 | # who have received LLITNs |  |  |  |  |  |  |  |  |  |  |  |  |  |
| *iv.* | *Total # 3rd trimester ANC visits (>=28 weeks) per month* |  |  |  |  |  |  |  |  |  |  |  |  |  |
| 11 | # of 3rd trimester ANC visits protected against tetanus (at least TT2) per month |  |  |  |  |  |  |  |  |  |  |  |  |  |
| 12 | # of 3rd trimester ANC visits receiving IPT2 (or more) per month |  |  |  |  |  |  |  |  |  |  |  |  |  |
| D | **HIV Testing & Treatment (PMTCT)** |  |  |  |  |  |  |  |  |  |  |  |  |  |
| *v.* | *Total # ANC visits at facility per month  (= new + revisit)* |  |  |  |  |  |  |  |  |  |  |  |  |  |
| 13 | # of pregnant women whose HIV status is known |  |  |  |  |  |  |  |  |  |  |  |  |  |
| 14 | # of HIV +VE pregnant women |  |  |  |  |  |  |  |  |  |  |  |  |  |
| 15 | # of life births from HIV+VE mothers |  |  |  |  |  |  |  |  |  |  |  |  |  |
| 16 | # of babies born to HIV+ve Mothers alive at 6 months & HIV-VE (PCR done) |  |  |  |  |  |  |  |  |  |  |  |  |  |
| 17 | # of babies born to HIV+ve mothers alive at 12 months & HIV-VE (PCR Done) |  |  |  |  |  |  |  |  |  |  |  |  |  |
| 18 | # of babies born to HIV+VE mothers alive at 18 months & HIV-VE (PCR/Antibody test) |  |  |  |  |  |  |  |  |  |  |  |  |  |
| 19 | # of HIV +VE pregnant women on preventive ARVs |  |  |  |  |  |  |  |  |  |  |  |  |  |
| E | **Community Linkages** |  |  |  |  |  |  |  |  |  |  |  |  |  |
| 20 | # of pregnant women referred to the health facility by community representative (e.g. by CHW, TBA) |  |  |  |  |  |  |  |  |  |  |  |  |  |

| **Members of the QI Team:**  Name: __________________________________________ Position: ____________________  Name: ­­­_________________________________________ Position: ____________________  Name: ­­­__________________________________________ Position: ____________________  Name:­ ­­__________________________________________ Position: ____________________  Name: ­­­__________________________________________ Position: ____________________  Name: ­­­__________________________________________ Position: ____________________  Name: ­­­__________________________________________ Position: ____________________  Name: ­­­__________________________________________ Position: ____________________ |
| --- |
