# Supplementary material for: Improving service uptake and quality of care of integrated maternal health services: the Kenya kwale district improvement collaborative
Source: BMC Health Serv Res. 2014 Sep 21;14:416. doi: 10.1186/1472-6963-14-416 (PMC4179240; doi:10.1186/1472-6963-14-416)
Supplement: Supplementary file 2 — Additional file 2: Change Package for Improving the Quality of Antenatal Care Services and Skilled Deliveries in Kwale, Kenya. (DOCX 397 KB) [file 12913_2013_3504_MOESM2_ESM.docx]

Change Package for Improving the Quality of Antenatal Care Services and Skilled Deliveries in Kwale, Kenya

Michael K Mwaniki^1,2^ , Sonali Vaid^1^, Isaac Mwamuye Chome^1^, Dorcas Amolo^1^, Youssef Tawfik^1^, Kwale Improvement Coaches ^3^

Michael K Mwaniki: [michael.kivkiv@gmail.com](mailto:michael.kivkiv@gmail.com)

Sonali Vaid: [sonali.vaid@gmail.com](mailto:sonali.vaid@gmail.com)

Isaac Mwamuye Chome: [ichome@urc-chs.com](mailto:ichome@urc-chs.com)

Dorcas Amolo: [dorcas.amolo@gmail.com](mailto:dorcas.amolo@gmail.com)

Youssef Tawfik: [ytawfik@phcpi.org](mailto:ytawfik@phcpi.org)

Kwale Improvement Coaches: [kwalehealthdirector13@gmail.com](mailto:kwalehealthdirector13@gmail.com)

May 2014

**Acknowledgements**

DISCLAIMER

The contents of this report are the sole responsibility of University Research Co., LLC (URC) and do not necessarily reflect the views of the United States Agency for International Development or the United States Government.

The authors would like to acknowledge all the people who contributed to the development of this change package, especially the quality improvement teams and their coaches whose work is disseminated in this document. This work would not have been completed without the support of the District Medical Officer of Health - Kwale, the entire District Health Management Team, the Provincial Health Management Team, and the Ministry of Health. The authors also acknowledge the contributions of Faith Mwangi-Powell and Esther Kahinga in editing the final document.

The work described was made possible by the generous support of the American people through the United States Agency for International Development (USAID) and carried out under the USAID Health Care Improvement (HCI) Project, managed by University Research Co., LLC (URC) under the terms of Contract Number GHN-I-03-07-00003-00. Consolidation and refinement of the change package was carried out under the USAID Applying Science to Strengthen and Improve Systems (ASSIST) Project, managed by URC under the terms of Cooperative Agreement Number AID-OAA-A-12-00101. The authors thank the HCI and ASSIST teams in Kenya and Bethesda for providing technical support throughout the project and in the development of the change package.

The work of the USAID ASSIST Project is supported by the American people through the USAID Bureau for Global Health, Office of Health Systems. URC’s global partners for USAID ASSIST include: EnCompass LLC, FHI 360; Harvard University School of Public Health; Health Research, Inc.; Initiatives Inc.; Institute for Healthcare Improvement; Johns Hopkins University Center for Communication Programs; and Women Influencing Health Education and the Rule of Law, LLC. For more information on the work of the USAID ASSIST Project, please visit [www.usaidassist.org](http://www.usaidassist.org) or contact [assist-info@urc-chs.com](mailto:assist-info@urc-chs.com).

**Recommended citation**

Mwaniki M, Sonali V, Chome I, Amolo D, Tawfik Y, Kwale Improvement Coaches. 2014. Change package for improving the quality of antenatal care services and skilled deliveries in Kwale, Kenya.

Table of Contents

List of Figures ii

List of Acronyms and Abbreviations ii

1 INTRODUCTION 1

1.1 Background 1

1.2 Formation of improvement teams 4

1.3 Harvesting of change ideas 4

1.4 Intended distribution 4

2 RESULTS 5

2.1 Developing the change package 7

3 CHANGE CONCEPTS AND IDEAS 8

3.1 ANC coverage 8

3.2 Quality of antenatal care 9

3.3 Community linkages 10

3.4 Skilled deliveries 11

3.5 High level management-related changes 12

3.6 Prevention of mother-to-child transmission of HIV 12

4 DETAILED HOW TO GUIDE 13

4.1 Antenatal care services coverage 13

4.1.1 Integrating ANC services into outreaches 13

4.1.2 Change concept: Improved quality of ANC services at the facility 14

4.1.3 Change concept: Re-designation and working with TBAs. 15

4.1.4 Change concept: Community involvement and dialogue 16

4.1.5 Change concept: Use of mobile phones for follow-up of pregnant women 17

4.2 Quality of antenatal care 18

4.2.1 Change concept: Improve access to laboratory services by pregnant women in ANC 18

4.2.2 Change concept: Ensure continuous supply of haematinics 20

4.2.3 Change concept: Ensure all pregnancy women have their blood pressure measured and documented. 20

4.2.4 Change concept: Improve IPT adherence 21

4.2.5 Change concept: create good relationship between the health care workers and the community 21

4.3 Skilled deliveries 21

4.3.1 Change concept: Improvement of infrastructure for delivery 21

4.3.2 Change concept: Increasing awareness on safe motherhood to pregnant women and other community members 22

4.3.3 Change concept: Offering mother-friendly services to pregnant mothers. 23

4.3.4 Change concept: Improving access to delivery services at the facility. 23

4.3.5 Change concept: Re-designation and working with TBAs 24

4.3.6 Change concept: Public private partnerships 25

4.4 Prevention of mother-to-child transmission of HIV 25

4.4.1 Change concept: Increase awareness of HIV in pregnant women 25

4.4.2 Change concept: Increase accessibility and utilization of quality PMTCT services 26

4.4.3 Change concept: Improved commodity management of ARVs and related items 27

4.5 High level management-related changes 27

4.5.1 Change concept: Use of data to inform staffing needs 27

4.5.2 Change concept: Coaching and facilitative supervision 27

4.5.3 Change concept: public-private partnership 28

4.6 Community linkages 28

4.6.1 Change concept: To empower the community to demand and own safe motherhood services in the health facilities to improve their health. 28

## List of Figures

Figure 1: Where women in Kenya give birth 1

Figure 2: Model for improvement with the plan-do-study-act cycle 3

Figure 3: Percentage of pregnant women completing 4+ANC visits and skilled deliveries 5

Figure 4: Increasing antenatal care coverage in Kwale, January 2011-August 2012 6

Figure 5: Pregnant women receiving recommended care during ANC visits 6

## List of Acronyms and Abbreviations

ANC Antenatal care

ASSIST USAID Applying Science to Strengthen and Improve Systems Project

CBOs Community-based organizations

CHEW Community health extension worker

CHWs Community health workers

CORPs Community-owned resource persons

DBS Dried blood spot

DHC Dispensary Health Committee

DHIS District Health Information System

DHMT District Health Management Team

DMLT District Medical Laboratory Technologist

DOT Directly observed treatment

GOK Government of Kenya

HCI USAID Health Care Improvement Project

HCWs Health care workers

IPT Intermittent preventive therapy

Ksh Kenyan shilling

LMP Last menstrual period

OJT On-the-job training

PDSA Plan, Do, Study, Act

PHMT Provincial Health Management Team

PHT Public health technician

PMTCT Prevention of mother-to-child transmission

QIT Quality improvement team

TBAs Traditional birth attendants

SP Sulfadoxine-Pyrimethamine

URC University Research Co., LLC

USAID United States Agency for International Development

USD US dollar

# INTRODUCTION

## Background

The World Health Organization defines maternal morbidity and mortality as illness or death during pregnancy or childbirth, or within two months of the birth or termination of a pregnancy. The fifth Millennium Development Goal aims to reduce the maternal mortality ratio by 75% between 1990 and 2015. In Kenya, maternal mortality remains high at 488 maternal deaths per 100,000 live births (Kenya Demographic and Health Survey 2008-2009). While this is below the Sub-Saharan average of 640 deaths per 100,000, Kenya has experienced a slow progression in maternal health.

Most maternal deaths in Kenya are due to causes directly related to pregnancy and childbirth, unsafe abortion, and obstetric complications such as severe bleeding, infection, hypertensive disorders, and obstructed labor. Others are due to causes such as malaria, diabetes, hepatitis, and anaemia, which are aggravated by pregnancy.

While approximately 92% of women giving birth received some antenatal care in 2010, only 47% had the recommended four or more antenatal care visits. Over half (56%) of Kenyan women delivered at home, with home births being more common in rural areas, and only 44% of births were assisted by a health care professional (doctors, nurses and midwives) (see Figure 1). Rates of antenatal care and skilled birth attendance have declined over the past 10 years, particularly among the poor.

Maternal morbidity and mortality in Kenya results from the interplay of social, cultural, economic, and logistical barriers, coupled with a high fertility rate and inadequate and under-funded health services. Strengthening the health system and improving quality of health care delivery is pivotal to reversing the trend of high maternal morbidity and mortality.

**
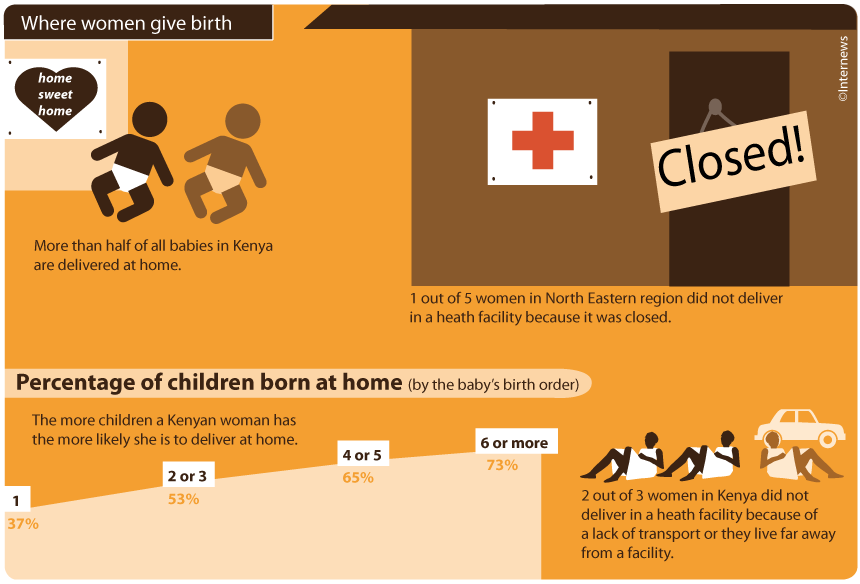
****Figure 1: Where women in Kenya give birth**

**1.2 USAID Health Care Improvement Project**

The USAID Health Care Improvement Project (HCI) was a global project funded by the United States Agency for International Development (USAID) and implemented by University Research Co., LLC (URC). Guided by the vision that health care can be significantly improved by applying scientifically demonstrated quality improvement (QI) methods, the project assisted country health authorities and implementing partners to expand programs to improve outcomes in child health, maternal and newborn care, HIV and AIDS, TB, malaria and reproductive health.

In Kenya, HCI began operations in 2009 with initial focus on orphans and vulnerable children. In January 2011, the project expanded to include maternal health, with specific focus on antenatal care services and skilled deliveries. HCI collaborated with the Ministry of Health to improve maternal health in Kwale District using quality improvement methods. A simple definition of health care quality is ‘*the degree to which health services for individuals and populations increase the likelihood of desired outcomes and are consistent with current professional knowledge.’* Kwale District was selected by the Ministry of Health for the intervention.

1.3 The science of improvement

Modern improvement approaches offer methods for overcoming common barriers to quality care, such as lack of awareness of standards, low provider competence, poor organization of care, and lack of motivation or rewards for quality. Improvement methods improve processes of care based on four principles: 1) understanding and focusing on client needs; 2) understanding how processes of care function within the system; 3) testing changes and using data to measure results; and 4) engaging teams of managers, service providers and community stakeholders in improvement.

The teams are called QI teams when formed at the health facility level, though large health facilities also form work improvement teams at departmental level.

In quality improvement work, teams analyze their own systems and processes of care, identify and test changes in the organization of care that may result in improved quality and efficiency, and measure the effect of changes through data. A central tenet of improvement is that local health system participants have the profound knowledge of their systems and are best positioned to identify, test, and implement improvements (change ideas) to achieve the highest quality of care possible in their setting. Engaging teams of providers in regular analysis of locally collected data and continuous quality improvement helps foster a culture of quality that contributes to health worker motivation.

In order to rapidly spread what different improvement teams learn as they test and implement change ideas, an improvement collaborative^1^ is often organized. An ‘improvement collaborative’ is a shared learning system that brings together a large number of teams to work together to rapidly achieve significant improvements in processes, quality, and efficiency of a specific area of care, with intention of spreading these methods to other sites. Improvement collaboratives seek to adapt and spread existing knowledge to multiple sites. The existing knowledge may consist of clinical practices based on scientific evidence, proven practices that are widely considered as ‘good’ or even ‘best,’ or any other changes to the existing way of doing things that have been shown to result in better health care. Such knowledge is the collaborative’s ‘change package,’ or the changes in processes and organization of care that the collaborative seeks to introduce, refine, and spread. Collaboratives are intended as a time-limited improvement strategy, typically achieving significant results in 9-18 months, although improvements are often seen much earlier.

^1^ USAID Health Care Improvement Project. 2008. The Improvement Collaborative: An Approach to Rapidly Improve Health Care and Scale up Quality Services. Published by the USAID Health Care Improvement Project. Bethesda, MD: University Research Co., LLC (URC). Available at: https://www.usaidassist.org/resources/improvement-collaborative-approach-rapidly-improve-health-care-and-scale-quality-services.


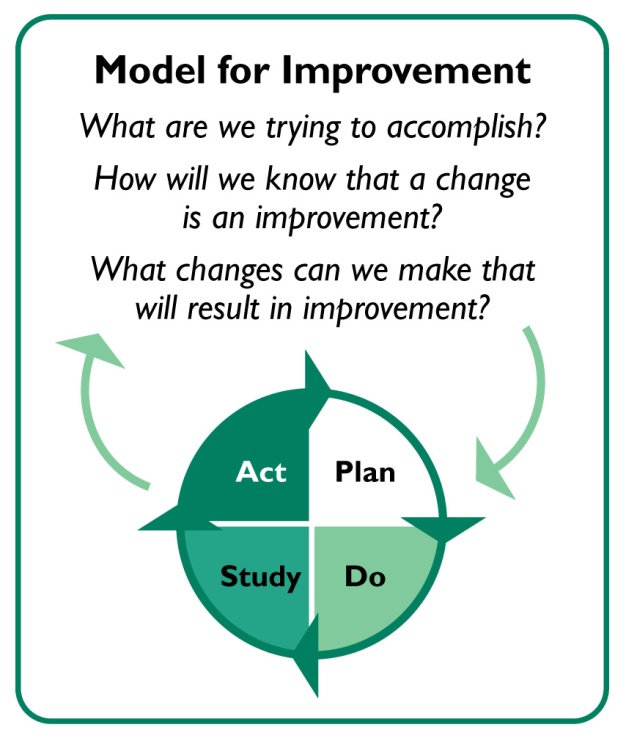
Teams test changes by applying an improvement or change model. The model used in most HCI-supported collaboratives is shown in Figure 2 and is known as the Model for Improvement, incorporating the plan-do- study-act (PDSA) cycle, described in *The Improvement Guide.^2^* In this model, a change believed likely to yield improvement is proposed. However, whether it will yield an improvement or not is a hypothesis that needs to be proved or disapproved. A plan is developed for testing the change, the plan is implemented, and the effect of that test is studied to see whether the change did in fact yield the improvement expected. Changes that yield the expected results are adopted while those that do not are either modified or abandoned.

Figure 2: Model for improvement with the plan-do-study-act cycle

**1.4 The Kwale improvement collaborative**

Kwale District is one of the resource-constrained counties in Kenya with an estimated population of about 160,000 people with very low literacy levels and low health indicators. The district includes 21 health facilities.

In 2011, maternal mortality in Kwale District was estimated to be 590-700 deaths/100,000 live births, and deliveries by skilled birth attendants were less than 30% of the deliveries in the district, with a similar percentage of pregnant women completing at least four antenatal care (ANC) visits.

The leading causes of maternal death then were:

- Severe bleeding (mostly bleeding after childbirth)
- Infections (usually after childbirth)
- High blood pressure during pregnancy (pre-eclampsia and eclampsia)
- Unsafe abortion.

Most of the mortality could have been avoided if proper care had been provided to the mothers during the antenatal period, delivery conducted by a skilled health attendant, and post-delivery care given. The poor coverage of antenatal services and skilled deliveries in Kwale District informed the choice of the district for this work.

The Kwale collaborative improvement project began in January 2011 with support from the USAID Health Care Improvement (HCI) Project in partnership with the then two Ministries of Health in Kenya – the Ministry of Public Health and Sanitation and the Ministry of Medical Services.

^2^ Langley G.J., Nolan K.M., Nolan T.W., Norman C.N., Provost L.P. 1996. *The Improvement Guide: A Practical Approach for Enhancing Organizational Performance*. San Francisco: Jossey-Bass.

To address the challenges that had been noted in Kwale, three improvement aims were developed as follows:

- Improve the quality and utilization (effectiveness) of antenatal care services (evidence-based, high-impact interventions)
- Improve the quality and utilization of institutional childbirth services (given low skilled delivery rate)
- Strengthen community-facility linkages, continuity of care, and coverage of ANC and skilled care by improving linkages between community health workers, clients, and maternities

## Formation of improvement teams

The situation analysis, development of indicators to measure improvement, and involvement of key stakeholders was done between February and April 2011. The District Health Management Team (DHMT) members were trained on quality improvement principles, and each member was assigned to 2-3 facility teams to coach. Quality improvement teams (QITs) were formed in 20 government-managed and one faith-based health facilities. Each health facility had 7-12 people drawn from members of the community that were considered key in the fight against maternal mortality and included health care workers, traditional birth attendants (TBAs), community health workers (CHWs), provincial administration (chief/assistant chief), and spiritual leaders. The QITs in these facilities met regularly to analyze their data, identify gaps, find the root cause, identify change ideas, and test them to address the gaps.

## Harvesting of change ideas

The change ideas were monitored by HCI staff to see whether they led to an improvement or not. In collaborative improvement, each team tests changes using the plan-do-study-act cycle and shares their results with other teams. In this way, the effective changes can be scaled up while ineffective ones are either discarded or improved upon and tested again.

The third learning session of the project was held August 7-8, 2012, with 20 health facilities sharing their work. Through poster galleries, plenary sessions, and group activities, QITs shared the change ideas they had tested.

The participants included representatives of the QITs, health workers, administrative officers, community members, DHMT members from Kwale, Kinango and Msambweni districts, HCI staff, and representatives from the provincial health offices and the Ministry headquarters.

## Intended distribution

The Ministry of Health in Kenya is keen on rolling out quality improvement across all its service delivery points. This change package is specifically intended to be a useful resource for communities, health care workers, health managers as well as leaders and policy makers who seek to improve the health outcomes of pregnant women and thus reduce their morbidity and mortality. It is also useful for district and facility health managers who may be tasked with implementing quality improvement activities at their service delivery points in line with the Ministry of Health quality strategy.

The ideas that were ranked highly were found to be effective and therefore ready for implementation on a large scale, while those with moderate to low scores were to be adjusted and tested further on a small scale to see whether they would lead to an improvement or not.

# RESULTS

Improvement was monitored through regular collection and analysis of data from primary registers in the facility. The same indicators were also monitored through the national health information management system through the District Health Information System (DHIS) reporting system.

Tremendous improvement results were realized in the collaborative by its completion in August 2012. Data compiled by the DHMT from the primary facility registers showed that the percentage of pregnant women delivered by skilled birth attendants increased from 30% in January 2011 to an average 47% of the estimated number of pregnant women in the district by August 2012 (see Figure 3). In the same period, the percentage of pregnant women completing at least 4 ANC visits increased from 37% to 57% of the estimated number of pregnant women in the district. The percentage of pregnant women starting ANC in first trimester also increased from 7% to 21% in the same period (see Figure 4).

Figure 3: Percentage of pregnant women completing 4+ANC visits and skilled deliveries

Figure 4: Increasing antenatal care coverage in Kwale, January 2011-August 2012

On the quality of ANC services, all women receiving ANC services in the district had their blood pressure measured during each visit as opposed to only 32% before the start of the collaborative improvement intervention in Kwale. More than 80% of pregnant women attending ANC had their hemoglobin level measured and their blood group determined and recorded. More than 90% of those attending ANC received regular supply of haematinics (iron supplements) as opposed to 23% at the beginning of the project. (See Figure 5.)

Figure 5: Pregnant women receiving recommended care during ANC visits

Community-to-facility linkages were strengthened in Kwale District as a result of the improvement work. For example, the number of referrals for facility care by community representatives (CHWs and TBAs) increased in number from 13 in January 2011 to 75 in August 2012.

Other achievements included the opening of three new laboratories and renovation of delivery rooms in the district with the help of various stakeholders.

## Developing the change package

For over 20 months the quality improvement teams had innovated and tested many change ideas geared towards:

- Expanding ANC coverage
- Improving the quality of ANC services
- Strengthening community-to-facility linkages for continuity and better coordination and uptake of care
- Increasing utilization of skilled maternity childbirth services

These change ideas were documented and monitored using defined indicators to see whether they led to improvement or not.

At each learning session, representatives of the QITs shared the change ideas they had tested and the results realized. At the third learning session, these ideas were consolidated and later on redistributed to each QIT. The QITs identified those that they tested as a team, made corrections for those that were not correctly described, and added any others that were missing from the consolidated list.

A harvest meeting was held October 30-31, 2012 with representatives from the QITs, DHMT, and USAID HCI in Mombasa. During this meeting, the change ideas were classified into change concepts and ranked. Change ideas were gathered for the four areas listed above: increasing ANC coverage, increasing quality of antenatal care, strengthening community-facility linkages, and utilization of skilled deliveries. In addition, change ideas were harvested in two other areas: management-related changes that had an overall effect on care quality and availability and changes related to preventing mother-to-child transmission of HIV (PMTCT).

Ranking was based on four parameters:

- Evidence from the pilot - how many sites tested it
- Simplicity
- Scalability
- Relative importance (its contribution to the results achieved)

Each parameter had 5 as the highest score and 1 as the lowest; therefore the maximum score for an idea was 20 and minimum 4. The ranking enabled identification of ideas that could be scaled up to other areas, those that need further refining and retesting, and those that may not be worth implementing.

The participants then described in detail how each change idea was tested or implemented; this detailed “how to” guide is presented in section 4 of this report.

# CHANGE CONCEPTS AND IDEAS

## ANC coverage

| **Problem/Opportunity** | **Change concept** | **Change ideas** |
| --- | --- | --- |
| Many women go to TBAs for advice and “massage” early in pregnancy and then attend ANC late | Re-designation and working with TBAs | Re-designation of TBAs to ANC supporters and birth companions |
|  |  | Involvement of TBAs and community health workers in follow up of mothers who did not show up for ANC appointments |
|  |  | Making of a referral form to be used by TBAs in referring expectant mothers to the facility |
|  |  | Provision of non-monetary incentives to TBAs who bring mothers to facility for ANC and delivery (praising them/formal recognition) |
| Inaccessibility of ANC services by some women due to long distance | Integrating ANC into outreaches | Conducting outreaches on weekend when more clients are available and staff available also |
|  |  | Making examination of mothers in outreach sites more acceptable and comfortable, for instance, through provision of examination couches and private space for examination |
|  |  | Task shifting: Teaching community health workers how to perform some nursing tasks, e.g., taking vital signs so as to free the health worker to attend to ANC mothers during the outreaches |
|  |  | Involvement of stakeholders to support outreaches in terms of transport and lunch for health workers, e.g., APHIA Plus |
| Ignorance on importance of starting ANC early and also retrogressive cultural practices | Community Involvement and Dialogue | Regular community dialogue meetings with community-owned resource persons (CORPs) and other community groups |
|  |  | Involvement of the community leaders in planning outreaches, e.g., chiefs, village elders |
|  |  | Use of women groups in mobilization for outreaches. |
|  |  | Formation of ANC support groups |
| Poor quality of services with long waiting time | Improved quality of ANC services at the facility | Active screening of pregnancy among all women of reproductive age groups attending other services (outpatient, immunization services for older child, etc.), closing the loopholes for missed opportunities |
|  |  | Reducing waiting time through integrating of all ANC services into one room, and process mapping to eliminate unnecessary steps |
|  |  | In-depth staff discussion on feedback from the community to help change staff attitudes and enhance provider-client-community communication and improve attitude towards pregnant women |
| Few mothers completed 4 ANC visits | Use of mobile phones for follow-up of ANC mothers | Health workers mobile phone numbers given to traditional birth attendants so that they can call the health workers anytime they have a client to bring to the facility for ANC visits |
|  |  | Acquisition of a dedicated facility phone used to remind mothers of their ANC appointments and birth preparedness |

## Quality of antenatal care

| **Problem/Opportunity** | **Change concept** | **Change ideas** |
| --- | --- | --- |
| Lack of haematinics in health facilities and low uptake of haematinics among pregnant women | Ensure continuous supply of haematinics | Due to unreliable supply of haematinics by the Government, facilities set aside funds to purchase ferrous sulphate and mebendazole for pregnant women |
|  |  | Improved documentation in ANC registers |
|  |  | Improved commodity management to ensure stocks are re-ordered before they completely run out |
| Low uptake of intermittent preventive therapy (IPT) among pregnant women | To improve adherence to IPT | Implementation of directly observed treatment (DOTS) policy for IPT |
|  |  | Integration of ANC services into one room |
| Few pregnant women had their blood pressure measured and documented | Ensure all pregnant women have their blood pressure measured and documented | Lobbying partners and stakeholders to purchase blood pressure machines |
|  |  | Improved documentation of blood pressure measurements in ANC registers |
| Low coverage for complete ANC profile among pregnant women and CD4 measurement for HIV+ pregnant women | To improve access to laboratory services by ANC mothers | Integrating laboratory services in outreaches |
|  |  | Collecting specimen from ANC clients and taking them to the nearest facility where lab services for ANC profile are available |
|  |  | Collecting specimen from ANC clients and taking them to the district hospital for CD4 measurement for those that are HIV-positive |
|  |  | Collecting specimen from outreach sites and taking them to the facility for testing for the facilities that were unable to integrate lab services in their outreaches |
|  |  | Liaising with the laboratory technologist at a neighboring facility who would visit the dispensary on particular days of the week and do the ANC profiles (Sharing of laboratory personnel) |
|  |  | Raising of funds by Dispensary Health Committee/QIT to purchase laboratory equipment/reagents for start-up and maintenance of basic laboratories |
|  |  | Lobbying stakeholders to provide equipment for starting laboratories in facilities without laboratory services, e.g., Kenya Commercial Bank, politicians |
|  |  | Mothers who could not afford to pay for ANC profile at once were allowed to pay in installments or had the fee waived |
|  |  | Employing a lab technologist from the community using facility improvement funds where a laboratory is available but no Government of Kenya personnel to run it |

## Community linkages

| **Problem / Opportunity** | **Change Concept** | **Change Idea** |
| --- | --- | --- |
| Low utilization of ANC services by the community | Empower the community to demand and own safe motherhood services in the health facilities to improve their health | Involvement of the community leaders in planning outreaches e.g., chiefs, village elders, etc. |
|  |  | Re-designation of TBAs to birth companions; they now make sure pregnant women in their villages attend early ANC, complete at least 4 ANC visits, and accompany them to the dispensary to deliver |
|  |  | Provision of incentives to TBAs to bring mothers to facility for ANC and delivery, e.g., being given preference in service delivery instead of queuing |
|  |  | Involving TBAs and CHWs in follow-up of mothers who don’t show up for their ANC clinic appointments |
|  |  | Having regular community dialogue meetings with CORPs and other community groups |
|  |  | Use of women groups in mobilization for outreaches |
|  |  | Making of a referral form to be used by TBAs in referring expectant mothers to the facility |
|  |  | Formation of ANC support group which upon delivery the mother graduates to breastfeeding support group. In the group, the mothers meet regularly to discuss issues related to safe motherhood, including having a birth plan. |

## Skilled deliveries

| **Problem/ Opportunity** | **Change concept** | **Change idea** |
| --- | --- | --- |
| Some women preferred to deliver at home due to lack of privacy in some facilities | Improvement of infrastructure for delivery | Improved privacy of delivery rooms |
|  |  | Identifying and setting aside rooms to be used as delivery rooms exclusively |
|  |  | Lobbying for funds and writing proposals to partners to support in construction/ renovation of delivery rooms |
| Ignorance on the dangers of home deliveries among women and the community | Increasing awareness on safe motherhood to pregnant women and other community members | Having regular community dialogue meetings with CORPs and other community groups |
|  |  | Having regular health talks at the facility in the morning before start of services |
| Poor attitude of health workers evidenced by mistreatment of women during delivery | Positive change of staff attitude towards pregnant women | On-the-job training of health workers on good clinical management of patients |
|  |  | Staff meetings to discuss issues concerning patient management |
|  |  | Individual counseling of health workers who were reported to have a negative attitude towards clients |
|  |  | A system for rewarding and recognition of staffs who perform well |
| Women deliver at home because they cannot access a health facility especially at night | Increasing access to delivery services | Offering 24-hour coverage for delivery services |
| TBAs are trusted by pregnant women and many prefer to be delivered by them at home, instead of health workers at the health facilities | Working with TBAs | Provision of incentives to TBAs to bring mothers for ANC and delivery |
|  |  | Health workers mobile phone numbers given to TBAs so that they can call the health worker when they have a client to bring to the facility |
|  |  | Re-designation of TBAs as birth companions |
|  |  | Formation of TBA support groups and registering them as community- based organizations (CBOs) |
|  |  | Taking the TBAs through orientation package on safe motherhood |
|  |  | Attending to referrals from TBAs and other CORPs promptly to foster the importance of their contribution |
|  |  | Where possible attending to CORPs promptly when they present unwell at the facility |
|  |  | Making standard referral forms used by TBAs in referring expectant mothers to facility |

## High level management-related changes

| **Problem/ Opportunity** | **Change concept** | **Change idea** |
| --- | --- | --- |
| Rally management support for improvement work across the district | Use of data to inform staffing needs | Improved staff management based on needs as a result of collecting data on staffing and staff performance over a period of time |
| Rally management support for improvement work across the district | Coaching and facilitative supervision | Improved/enhanced communication and supervision between the frontline health workers and the DHMT due to regular coaching and mentorship |
| Lack of proper infrastructure and equipment in the facilities | Public-private partnership | Writing of proposals to donors to assist in equipment and infrastructure development of facilities |

## Prevention of mother-to-child transmission of HIV

|  | **Change Concept** | **Change idea** |
| --- | --- | --- |
| Ignorance on the importance of testing for HIV especially among pregnant women | Increase awareness of HIV in pregnant mothers | Regular community dialogue meetings on importance of testing for HIV and benefits of PMTCT |
|  |  | Regular health education sessions at the facility |
|  |  | Encouraging male spouses to accompany their wives to antenatal clinic |
| PMTCT services were underutilized and only available in a few facilities. | Increase accessibility and utilization of quality PMTCT services | Decentralization of PMTCT sites to all facilities in the district |
|  |  | Laboratory networking: health workers collect CD4 and dried blood spot (DBS) samples at the facilities and take it to Kwale District Hospital and KEMRI Laboratories for analysis |
|  |  | On-the-job training on how to collect CD4 and DBS samples and transportation |
| Perennial stock out of commodities | Improved commodity management of ARVs and related items | Redistribution of drugs, test kits, reagents, equipment, and non-pharmaceuticals from facilities with excess to those experiencing stock-outs |

# DETAILED HOW TO GUIDE

## Antenatal care services coverage

### Integrating ANC services into outreaches

| **Change idea** | **How to guide** | **Notes on level of evidence from the rating** |
| --- | --- | --- |
| Conducting outreaches on weekend when more staff were available | This was tried in facilities with dire staff shortage that could not leave the facility uncovered during the week days. The staff deliberated and agreed to do the outreaches during the weekend. Funding was allocated to facilitate the outreaches from the Health Sector Strategic fund supported by the health management committees. In one facility, Saturday was the market day, hence the turn-out for ANC services at the outreach site was also high on Saturdays. | Tested in only three facilities. Overall evidence was low/poor for the institutionalization and sustainability of this change idea. |
| Making examination of mothers in outreach sites more acceptable and comfortable, e.g., examination couch, private space for examination | The community was involved in identification of the outreach site, and identification of a place (classroom /enclosed room) to offer privacy for ANC clients. In some cases, the community raised funds to purchase examination couch through individual contributions. In others, the facility transferred the extra examination couches not in use at facility to these community ANC examination venues. | Tested in 9 sites. Scored 13/20. Good evidence for application of this change idea. |
| Task shifting—teaching community health workers how to perform some nursing tasks, e.g., taking vital signs so as to free the health worker to attend to ANC mothers during the outreaches | CHWs already trained both during recruitment and “on job” to assist at respective facilities with tasks likes weighing and taking vital signs. Staffs discussed with the CHWs to help with similar activities during the outreaches so that they can have more time to attend to ANC mothers. | Tested in 9 sites. Scored 16/20. excellent evidence for application of this change idea where there are active and motivated CHWs. |
| Involvement of stakeholders to support outreaches in terms of transport and lunch for health workers (e.g., APHIA Plus) | The QITs & DHMT liaised with willing partners to support outreaches, especially in facilities with large catchment areas. A proposal was drafted and presented to the said partners who agreed to fund them. | Tested in 11 sites. Scored 15/20. Excellent evidence for application of this change idea where there are partners willing to support such services. May however lead to “donor dependence” |

### Change concept: Improved quality of ANC services at the facility

| **Change idea** | **How to guide** | **Notes on level of evidence from the rating** |
| --- | --- | --- |
| Active screening of pregnancy among all women of reproductive age groups attending other services (outpatient, immunization services for older child, etc.), closing the loopholes for missed opportunities | The QIT teams discussed areas of missed ANC opportunities especially failure to screen mothers and take proper history during outpatient attendances. It was agreed that LMP period should be part of standard history taking. The same was to apply during immunization clinic. | Rated very high with a score of 18/20. Easy to implement. This change idea is part of good clinical care. |
| Reducing waiting time through integration of all ANC services into one room; process mapping to eliminate unnecessary steps | All facilities conducted process mapping as part of the initial steps after formation of QIT. The teams then examined the weak points such as to and fro movements. ANC services that could be done in one room were consolidated. This led to reduction of overall time taken by clients. | Rated very high with a score of 18/20. Very easy to implement and scale up at any facility. |
| In-depth staff discussion on feedback from the community to help change Staff attitudes and enhance provider-client-community communication and improve attitude towards pregnant women | Feedback was obtained from:   - Staff-initiated dialogues during the community meetings to get open criticism on services and their attitudes - CHWs brought back to the QITs the feedback on what the community perceives about their attitudes - QIT community members give feedback on community perception on staff attitudes - Two facilities have suggestion boxes for clients to anonymously give feedback   The staffs then act on the feedback received during regular staff meetings, or emergency staff meetings for very urgent matters and during QIT meetings. | Rated very high with a score of 18/20. Easy to implement. Minimal monetary requirement. Should be part of routine facility management approaches. |

### Change concept: Re-designation and working with TBAs.

| **Change idea** | **How to guide** | **Notes on level of evidence from the rating** |
| --- | --- | --- |
| Re-designation of TBAs as ANC supporters and birth companions | All facilities were asked to identify and register TBAs within their catchment area (facility in-charge & PHT). This was a requirement by the PHMT; initiated mechanisms to register TBAs as CBOs or self-help groups. The TBAs then scheduled regular meetings attended by a member of the QIT (in-charge, CHEW, PHT). During these meetings, there were discussions on what role TBAs can play to facilitate safe motherhood. | Tested in 17 sites. Evidence for application very high. May require higher level management support. |
| Use of TBAs and community health workers in follow-up of mothers who don’t show up for ANC visits | Develop an inventory of TBAs and CHWs  Give list of ANC mothers in their respective catchment areas to the TBAs and CHWs for them to follow up and report on progress monthly.  During ANC clinics, the facility staff identify mothers who have failed to turn up for ANC re-visits and the village they come from. This information is then shared with the registered TBAs and CHW from that area to trace the mother and find out the reason the client did not come back on the scheduled visit. This information is then relayed back to the facility and necessary action taken. | Tested in 8 sites. Scored13/20. Requires a committed and motivated group of CHWs, TBAs and/or a Community Unit. |
| Making of a referral form to be used by TBAs in referring expectant mothers  to the facility | QIT decided to prepare forms to help track referrals from the community and also make it a part of the process of care. One of the QIT members drafted the letter which was reviewed and accepted by the team. This draft letter was piloted with a few copies to selected TBAs and CHWs. When the idea was seen to work, more copies were printed for all the available CHWs and TBAs. The facility management committee approved the funds for printing and photocopying. | Scored12/20. Viable ideas but challenge include literacy level, some clients may think the form comes with unreasonable incentives etc. |
| Provision of incentives to TBAs who bring mothers to the facility for ANC and delivery (praising them/formal recognition) | Some QITs agreed to design and print certificates to recognize TBAs with high referrals. These are presented during one of the regular TBA meetings or in a special event organized to honor those with highest referrals.  Verbal recognition of TBAs at any venue for their work in supporting ANC and hospital dispensaries. | Scored13/20. Non-monetary incentives like verbal recognition, courtesy and respect are routine and should be part of community engagement. Monetary incentives may not be sustainable and should be discouraged. |

### Change concept: Community involvement and dialogue

| **Change idea** | **How to guide** | **Notes on level of evidence from the rating** |
| --- | --- | --- |
| Community dialogue meetings with CORPs and others community groups | The QITs identified the known CORPs. Then a letter was drafted and sent to the CORPs through the chief inviting them to a meeting at the facility to discuss the challenges of ANC care and skilled delivery and formulate a way forward.  Other facilities discussed with the CORPs’ representatives to the facility management teams. The CORPs were tasked to discuss the issues around ANC with their respective communities. | Tested in 14 sites. Rated high with a score of 15/20. Excellent evidence for application of this change idea since many facility management teams already have community representation. Need to effectively use these people to improve services. |
| Involvement of the community leaders in planning outreaches e.g., chiefs, village elders | The QITs tasked the PHT who is also a QIT member to liaise with chiefs and village elders in planning for outreaches regularly.  In other sites the in-charge liaised with the chiefs and village elders to organize and plan for outreaches. | Tested in 14 sites. Rated highly with a score of 16/20. Requires an active and motivated PHT. |
| Giving health education to women groups | TBAs identified and formed a group and registered it as a CBO. They met regularly and also made contributions. Health talks on ANC and delivery were given during their meetings.  Existing non-TBA groups were identified, and health workers attended their meetings to give health talks and inform them of outreaches in their area. | This idea scored poorly and was hard to implement. It is may be useful to modify the idea such that representatives from such groups are targeted. |
| Use of ANC support groups to encourage pregnant women to complete 4 ANC visits and deliver in a health facility | ANC group existed before. The QIT team decided to strengthen this group. The group meets at the health facility and is spearheaded by the health worker. Clients were recruited on reporting for first ANC visit. They were given dates for ANC support group which occurs every fortnight. | About 6-10 mothers attend per visit. Most therefore get to attend a few visits before delivery. This was actively tested in just one facility. |

### Change concept: Use of mobile phones for follow-up of pregnant women

| **Change idea** | **How to guide** | **Notes on level of evidence from the rating** |
| --- | --- | --- |
| Health workers mobile phone numbers given to traditional birth attendants so that they can notify the health workers anytime they have a client to bring to the facility for  ANC visits or delivery | QITs decided that the HCWs should give their numbers to ANC mothers during the clinic so that they can notify/alert the health worker in-case of any complication or emergency. This would enable the facility to prepare in advance before the client even arrives. The clients could also call to find out if there is someone at the facility especially at night and weekends so that they do not waste unnecessary time. During ANC visit the client phone number is also captured by the health provider and entered into the ANC register for follow-up. | This was found to be effective especially in facilities not covered for 24 hours. Rated high with a score of 15/20. Easy to use. Minimal abuse of phone numbers ever reported. |
| Acquisition of a phone for the QI team used to remind mothers of their ANC appointments and birth preparedness | The QIT team approached a partner who bought them a phone. They then approached the facility management team which provided airtime of 500/= per month. During the ANC visits, the clients’ mobile numbers or those of people who can be used to reach them i.e. spouses, relatives, neighbours etc are recorded. Clients are reminded of their scheduled visit the Friday before the week for the visit and further reminded within the week (Monday) | Tested in just one facility. Scored at 8/20-fair. Not costly to maintain spends less than 500/ksh per month roughly 6USD. Response from clients good. It advisable to test this idea in more facilities before scaling up on a large scale. |

## Quality of antenatal care

### Change concept: Improve access to laboratory services by pregnant women in ANC

| **Change ideas** | **How to guide** | **Notes on level of evidence from the rating** |
| --- | --- | --- |
| Integrating laboratory services in outreaches | Incorporate the lab personnel in outreach services.  Inform the community that ANC profile will be done at the outreach site. Assemble the necessary equipment’s and reagents and arrange for transport for the equipment’s to the site. ANC profile is done on site and results given. | Scored 9/20. Was significant but not simple to implement and scale up. When the lab person goes for outreach, lab services stall at the facility. |
| Collecting specimen from ANC clients and taking them to the nearest facility with lab services for ANC profile. | Identify a suitable facility with a lab to network with; set a particular day of the week for specimen collection. Proper collection, labeling and transportation are done. Collect results, record and disperse to the clients when they come for their next ANC visit. | This was effective in facilities without labs, though it needs a committed health worker and funds to cater for transport costs to implement. scored14/20 |
| Collecting specimen from ANC clients and taking them to the district  Hospital for CD4 measurement for those that are HIV+. | Identify a suitable lab to network with; set a particular day of the week for specimen collection. Proper collection, labeling and transportation are done. Collect results, record and disperse to the clients when they come for their next visit. | Was highly ranked and scored 19/20. Was0 also effective because of partners support in providing transport. Can be done by all facilities without lab if transport is available. |
| Collecting ANC profile specimen from outreach sites and taking them to facility for testing  for those unable to integrate lab services in the outreaches | Mobilize the community so that they are aware that ANC profile specimen will be collected, have all the correct specimen containers and carriers in place, collect the specimen, transport to lab. Collect results and record them. Disperse the results to clients during the next outreach at the site. | Rated poorly, scored7/20.  Unavailability of safe suitable specimen carriers and transport costs make it hard to implement. |
| Liaising with the Lab technologist at a neighbouring facility who would visit the dispensary on particular days of the week and do the ANC profiles**.** This was done by a facility with lab equipments but lacked the lab technologist. | Identify a suitable lab to network with; identify a room to act as a lab. Set specific date for ANC profile  Inform the staffs and arrange for his / her transport. Mobilize mothers for ANC services in the facility. | Scored 10/20.It is notable that implementing this change idea created a gap in the other facility. |
| Raising of funds by Dispensary Health Committee/QIT to purchase laboratory equipment/reagents | Facility staffs identified the need and made the estimates for the funds needed. Shared the idea with the DHC which accepted and budgeted for the reagents and equipment’s. | Was ranked average, scored 11/20. Important but not easy to implement due to the low incomes of most facilities. |
| New laboratories were opened with the help of stakeholders to increase the uptake of ANC profiles e.g. KCB, Politicians. | Proposals were written by both the DHMT and facility staffs (QITs) and delivered to various stakeholders. Follow up was done spearheaded by the DHMT. Partners who were able purchased the microscopes for the facilities for setting up laboratories. | Was ranked high, scored 15/20. Very important but not so many stakeholders responded positively. |
| Mothers who could not afford ANC profile at once were allowed to pay in installments or waived. | Assess and identify the needy mothers using a set criteria or by getting detailed information from community leaders. Those with insufficient funds were allowed to pay in installments spread throughout the ANC visits. Where resources allow, the very needy are waived. | Was ranked high scored 17/20 and encouraged women to start ANC in first trimester even if they did not have money to pay for ANC profile. |
| Facilities to employ a lab technologist from the community | Identify a lab technologist; liaise with the DMLT to ascertain qualifications, knowledge and skills. DHC budgeted for his/her payment and agreement signed with the lab technologist. | This was found to be useful but proper procedures must be followed. |

### Change concept: Ensure continuous supply of haematinics

| **Change idea** | **How to guide** | **Notes on level of evidence from the rating** |
| --- | --- | --- |
| Purchase of haematinics by facilities. | The QIT deliberated and established that the facility could self-sustain the procurement of certain commodities such as haematinics without relying on central supplies. They discussed this with the management which agreed to set aside funds from facility improvement fund (FIF) kitty to purchase haematinics regularly. | Scored 20/20. This was very effective and haematinics were cheap to purchase. Can easily be scaled up in most facilities and settings. |
| Improved documentation of ANC registers | Sensitization through on job training and Data review meetings on how to correctly fill the ANC register. The ANC register is filled in completely before the client leaves the room. | Scored 20/20, and was easy to implement. Regular supervision may be needed. |
| Improved commodity management | The nurses kept up to date haematinics bin cards. Keeping track of haematinics stocks on a monthly basis. Timely ordering of haematinics. | Easy to perform. Scored 15/20. |

### Change concept: Ensure all pregnancy women have their blood pressure measured and documented.

| **Change idea** | **How to guide** | **Notes on level of evidence from the rating** |
| --- | --- | --- |
| Partners were involved in purchasing of blood pressure machine | DHMT offices have an inventory of all equipment available in each facility and what requires replenishing  DHMTs solicited partners through talks that availed Blood Pressure (BP) machines for all facilities. | Scored12/20. Scaling up is an issue not many partners are willing to purchase basic equipments for facilities.( Thought to be the role of the government) |
| Improved documentation of blood pressure measurements in the ANC register. | In some facilities, BP was measured but not documented. A change of staff attitude and practices was thus necessary. The teams decided that the ANC register is to be filled before the mother leaves the room. On job training to the staffs on the ANC register. | This was highly effective and scored 20/20. It required minimal resources to implement and scale up. |

### Change concept: Improve IPT adherence

| **Change idea** | **How to guide** | **Notes on level of evidence from the rating** |
| --- | --- | --- |
| Implementation of DOTS policy for IPT | Educated the mothers on the importance of taking the Sulfadoxine-Pyrimethamine (SP) for IPT in the facility during the general health talk and the one on one counseling at the ANC room.  Provision of clean water and cups at point of dispensing SP for IPT (ANC room) so that the mother takes the SP for IPT as the health worker observes.  Availed the policy/guideline document on IPT for the staff and client to read at will. | Was ranked very high and is easy to implement. This is a government policy and all facilities have the capacity to implement it. |
| Integration of ANC services into one room | SP for IPT was given to the nurse attending ANC clients to dispense in the ANC room so that the mother does not have to queue again at the pharmacy. Clean water and cups were availed for the mothers to take the IPT as the health worker observed. | Was ranked very high, scored 20/20. |

### Change concept: create good relationship between the health care workers and the community

| **Change idea** | **How to guide** | **Notes on level of evidence from the rating** |
| --- | --- | --- |
| Change of staff attitude and practices | Health workers reviewed their attitudes and practices to clients on their own. They also got regular feedback on how the community views their attitude and practices during the QIT meetings. Efforts were made to change all the bad attitudes and practices that were not acceptable to the community. Regular feedback mechanisms are in place through QIT and DHC members who are from the community. | Was highly scored, 20/20, and found to be very effective and did not require any resources to implement. However a lot of good will is needed from the health workers. |

## Skilled deliveries

### Change concept: Improvement of infrastructure for delivery

| **Change idea** | **How to guide** | **Notes on level of evidence from the rating** |
| --- | --- | --- |
| Improved privacy of delivery rooms /setting aside rooms exclusively for deliveries | Rooms were identified and set aside exclusively for deliveries. Privacy was enhanced through having curtains in place, and restricting entrance to the rooms.  Some facilities wrote proposals to partners to assist them in building maternity rooms.  Others renovated rooms and used them as maternity rooms. | This scored 16/20. It was effective as utilization increased in those facilities that privacy was improved.  It was otherwise difficult to find stakeholders /partners who could fund building projects. Some of those that provided support did so but provided sub-standard structures |

### Change concept: Increasing awareness on safe motherhood to pregnant women and other community members

| **Change ideas** | **How to guide** | **Notes on level of evidence from the rating** |
| --- | --- | --- |
| Enhanced /improved communication between service providers and pregnant mothers | Health workers spent time with mothers during ANC visits and counseled them on individual birth plans, among other needed areas of counseling. The expectant mother freely asks any question and is answered satisfactorily and respectfully. | The clients felt appreciated and got the information well. The challenge is that the health worker takes long to deal with one client hence not very easy to implement effectively in facilities with few staff and a big catchment population. Ranked highly with a score of 19/20. |
| Having regular community dialogue meetings with CORPs and other community groups. | The QIT identified the known CORPs. Then a letter was drafted and sent to the CORPs through the chief inviting them to a meeting at the facility to discuss the challenges of ANC care and skilled delivery and formulate a way forward.  Other facilities discussed with the CORPs’ representatives to the facility management teams. The CORPs were tasked to discuss the issues around skilled deliveries with their respective communities. | Scored 17/20.  Community dialogue-resulted into improved relations between the community and institution.  Co-operation with Administration Officers was necessary. |
| Positive change on staff attitude towards treating pregnant women treating better | Capacity building through training  Staff meetings and individual counseling  Appreciating individual contributions to the team and rewarding of best- performing staff and department.  Health workers reviewed their attitudes and practices to clients on their own. They also got feedback on how the community views their attitudes and practices. Efforts were made to change all the bad attitudes and practices that were not acceptable to the community. Regular feedback mechanisms are in place through QIT and DHC members who are from the community. | Staff appreciated the need for change and they understood the impact it would have on health coverage in general.  This change idea was scored16/20 and can be scaled up at minimal cost. |
| Health talks at the facility in the morning before start of services. | HCWs and CHWs worked together to form duty rosters for which each of them had a particular day of the week that they would give health education at the facility in the morning before start of service. Methods of communication included dialogues and Education Through Listening. | This is easy to implement in all facilities as it does not involve any financial inputs. It is supposed to be part of the routine in all facilities. |

### Change concept: Offering mother-friendly services to pregnant mothers

| **Change ideas** | **How to guide** | **Notes on level of evidence from the rating** |
| --- | --- | --- |
| Provision of warm water bath, warm porridge, and hygiene pack to mothers after delivery. | Bench mark with TBAs on what other services they offer mothers to attract them and create a conducive environment for delivery, e.g., warm bath, back massage, warm drink after delivery, etc. Offer those hospitality services at the facility or allow the TBA or relative to offer those services to the mother as they deliver at the facility.  Sought funds from the facility management committee to implement these tasks which was granted and was also included in the Annual Work Plan to ensure sustainability.  Include cost (Ksh 50) of hygiene pack consisting of sanitary pads, toilet soaps, and bar soap in the client’s bill. This was tested at the district hospital. | Mothers appreciated the input. Regular funds are needed to sustain these services which were found to be attractive to mothers. Scored 16/20. |

### Change concept: Improving access to delivery services at the facility

| **Change idea** | **How to guide** | **Notes on level of evidence from the rating** |
| --- | --- | --- |
| Offering 24 hours coverage for deliveries. | Facilities made duty allocation, and each week a staff was put on night call to attend to mothers who came at night to deliver.  Health care workers moved into the staff quarters (where available) or rented near the facility where they could easily walk to the facility when needed even after regular working hours.  Casual labourers on night duty at the facility were also given the duty of informing the health care workers whenever a woman in labour came to the facility. | This was found to be an effective innovation which needed a lot of commitment from the health care workers.  Staff shortage, lack of staff houses, and insecurity made it difficult to initiate and maintain provision of 24 hours coverage in some facilities. Scored14/20. |

### Change concept: Re-designation and working with TBAs

| **Change idea** | **How to guide** | **Notes on level of evidence from the rating** |
| --- | --- | --- |
| Provision of incentives to TBAs to bring mothers to the facility for skilled delivery. | Health care workers scheduled regular talks /dialogues with TBAs on safe motherhood and dangers of home delivery. TBAs were given priority when they brought clients to the facility or even when it was themselves that come for treatment.  Certificates were printed and given to those who referred most mothers.  Verbal appreciation/recognition was always done each time a TBA referred a mother. | This was ranked highly, scored 20/20, and can be done by all facilities easily.  Some TBAs claimed monetary incentives which the facility could not provide.  Monetary incentives may not be sustainable and should be discouraged. |
| Health care workers mobile phone numbers were given to TBAs so that they could inform the HCWs in advance when bringing a client or ask for advice when in need. | HCWs shared their personal mobile numbers with TBAs and other CORPs during dialogue meetings. The TBAs inform the HCWs when they are referring/bringing a mother in labour to the facility for the HCW to prepare for their arrival especially during weekend and nights. | Scored18/20. Eased communication and improved staff preparedness for clients.  Challenge: odd hour calls and irrelevant calls, though this rarely happened. |
| Re-designation of TBAs to birth companions | All facilities were asked to identify and register TBAs within their catchment area (facility in-charge & Public Health Technician). This was a requirement by the provincial health management team. Initiate mechanisms to register themselves as CBO or self-help groups. They were to schedule regular meeting attended by a member of the QIT (in-charge, CHEW, PHT). During these meetings, there were discussions on what role they can play to facilitate safe motherhood.  The TBAs refer or escort mothers to the facility during delivery and can offer other services to them such as prepare warm bath and hot porridge for the mother after delivery. | This was effective and was scored 15/20. With support at the policy level, tremendous results can be achieved if rolled out in a large scale. Resulted into more active TBAs.  However some TBAs started demanding cash incentives. |
| Making of referral forms used by TBAs in referring expectant mothers to facility | QIT decided to prepare forms to help track referrals from the community and also make it a part of the process of care. One of the QIT members drafted the form which was reviewed and passed by the team. This draft referral form was piloted with a few copies to selected TBAs and CHWs. When the idea was seen to work more copies were printed for all the available CHWs &TBAs. The facility management committee approved the funds for printing and photocopying. | Scored12/20. Viable ideas but challenge includes literacy level, and also some clients may think the form comes with unreasonable incentives etc. It is worth noting that the national tool kit for CHWs include a referral form. |

### Change concept: Public-private partnerships

| **Change idea** | **How to guide** | **Notes on level of evidence from the rating** |
| --- | --- | --- |
| Stakeholders brought on board to improve the quality of services at facility and district level | The QIT identified the needs and conducted stakeholders mapping.  Drew a budget and wrote proposal for support in the relevant areas, e.g., building, repairs, furnishing and equipping.  Also identified stakeholders to help in providing trainings on various topics, even to fund educative visits to areas and facilities that had certain projects running that were of interest | Scored, 16/20.  Delivery rooms were built, capacity building done (both for staff and community representatives)  Involvement of stakeholders in the improvement of overall health status of the community, which has been appreciated by them (good relationship between stakeholders and community). |

## Prevention of mother-to-child transmission of HIV

### Change concept: Increase awareness of HIV in pregnant women

| **Change idea** | **How to guide** | **Notes on level of evidence from the rating** |
| --- | --- | --- |
| Regular community dialogue meetings on importance of testing for HIV and benefits of PMTCT | Community dialogue meetings were held through chief’s barazas, TBAs, religious gatherings, and other community gatherings where issues of HIV and PMTCT were discussed. | Scored17/20  Meetings already existed but QITs helped to activate and strengthen them.  Male partners’ perceptions on testing affects dialogue; e.g., when a partner is tested HIV-positive, the male partner concludes that he too is HIV-positive, hence see no need to take part in Community Dialogues. |
| Health education sessions at the facility | HCWs and CHWS worked together to form duty rosters for which each of them had a particular day of the week that they would give health education at the facility in the morning before start of service. | Scored19/20.  This is easy to implement in all facilities as it does not involve any financial inputs. |
| Male spouse involvement and couple testing | HCWs encouraged pregnant mothers to ask their husbands to accompany them when they came for ANC clinic. Small notes were given to the mothers to give to their spouses asking them to accompany their spouses in the next ANC visit. Women accompanied by their spouses were attended to first and their partners tested for HIV. In addition the male partner was offered other services such as simple medical check-ups, e.g., blood pressure measurement as an incentive. | Scored 9/20.  The turn-out from the male spouse was not good.  Male spouse assumed their HIV status based on his partner’s results; the need for actual testing needs to be encouraged.  This idea may be repackaged and tested again. |

### Change concept: Increase accessibility and utilization of quality PMTCT services

| **Change ideas** | **How to guide** | **Notes on level of evidence from the rating** |
| --- | --- | --- |
| Decentralization of PMTCT sites to all facilities in the district | All facilities in the district became PMTCT sites and the staff were trained and mentored through on-job training. Regular supportive facilitative was done and regular supply of antiretrovirals (ARV) was ensured. | Was found to be effective and scored highly 17/20, can be implemented on a large scale with proper training and supervision. |
| Laboratory networking: health workers collect CD4 and DBS samples at the facilities and take them to the District Hospital and KEMRI Labs for analysis. | Identify a suitable lab to network with; set up a particular day of the week for specimen collection. Proper collection, labeling and transportation are done. Collect results, record and disperse to the clients when they come for their next visit. | Scored 16/20.  However, the initiative was successful due to support by partners. Service currently supported by partners up to 75% of the total cost.  This can be implemented across many facilities easily when partner support is available. For sustainability, facilities have to include it in their Annual Work Plan. |
| On-the-job training on how to collect CD4 and DBS samples and transportation | HCWs that were well versed with the procedures visited other sites and showed their colleagues how to collect, label, and transport the samples. | Scored13/20.  Likewise success was largely attributable to substantive donor/partner support. |

### Change concept: Improved commodity management of ARVs and related items

| **Change ideas** | **How to guide** | **Notes on level of evidence from the rating** |
| --- | --- | --- |
| Redistribution of drugs, test kits, reagents, equipments and non-pharmaceuticals from facilities with excess to those experiencing stock-outs. | This was done by the DHMT in collaboration with the facilities through enhanced supportive supervision. Immediate actions to replenish stocks were done by redistribution, especially when there was delayed supply from the national level. | Was scored 18/20. With an active DHMT, this can be implemented successfully. |

## High level management-related changes

### Change concept: Use of data to inform staffing needs

| **Change ideas** | **How to guide** | **Notes on level of evidence from the rating** |
| --- | --- | --- |
| Improved staff management based on needs- informed decisions using data on work load and staffing needs | The DHMT used data from the facilities to know the workload of each facility. Re- allocation of staff was done to reflect the workload. Likewise in some facilities initially most staffs were out of training or upgrading courses. The DHMT redistributed staffs to ensure that several staffs from one facility could not all be out for training simultaneously. | Scored17/20. This was difficult sometimes due to an acute shortage of HCWs in the district. |

### Change concept: Coaching and facilitative supervision

| **Change ideas** | **How to guide** | **Notes on level of evidence from the rating** |
| --- | --- | --- |
| Improved/ enhanced communication and supervision from the DHMT | Each DHMT member was allocated 2-3 facilities to act as a coach. This entailed a much closer approach than routine supportive supervision. DHMT members would keep in touch with their teams through phone communication and scheduled on-site coaching visits.  This approach ensured that the DHMT members became members of the QITs and hence became more familiar with issues affecting the facility. | Scored 15/20. This approach ensured the DHMT overall was highly reactive to issues raised by the QITs leading to a better working environment. |

### Change concept: public-private partnership

| **Change ideas** | **How to guide** | **Notes on level of evidence from the rating** |
| --- | --- | --- |
| Writing of proposals to partners, e.g., local banks to assist in acquiring equipment and infrastructure. | The QITs identified some infrastructure and equipment challenges that were hindering delivery of services and discussed them with the DHMT. The DHMT then assisted the QITs to write proposals to selected stakeholders within the district. Some responded positively and constructed some infrastructure, while others purchased equipment. | This normally takes a long time, and it has to fit into the partners’ budget and area of interest.  Three laboratories were started due to these efforts.  Scored16/20. |

## Community linkages

### Change concept: To empower the community to demand and own safe motherhood services in the health facilities to improve their health.

| **Change concept** | **How to guide** | **Notes on level of evidence from the rating** |
| --- | --- | --- |
| Involvement of the community leaders in planning outreaches e.g., chiefs, village elders etc.  This fosters ownership for the outreaches from the community. | QITs identified the Key leaders and CORPs within their catchment area. They conducted planning meetings with the leaders on many areas that required improvement and discussed how the community could get involved both at the facility and during outreaches. | Highly rated.  This can be replicated in many areas easily. |
| Re-designation of TBAs to birth companions: they now make sure pregnant women in their villages attend early ANC, complete at least 4 ANC visits, and accompany them to the dispensary for delivery. | QIT did an inventory of TBAs within its catchment area  The TBAs were then encouraged to register themselves as CBOs and compete for projects with other CBOs rather than conducting deliveries at home.  Regular dialogue meetings were held with the TBAs to encourage them to actively refer any women coming to them for services at the nearest health facility. | Rated very high. Scored 20/20  Existence of active TBAs in the community is a prerequisite.  Referrals by TBAs increased. |
| Provision of incentives to TBAs to bring mothers to facility for ANC and delivery, e.g., being given preference in service delivery instead of queuing | Identification of active TBAs through the number of ANC referrals made.  Share feedback to stakeholders.  Recognize and reward.  Exemption of TBAs from user fees.  No queuing when they come to the health facility for services. | Rated very high with a score of 19/20  Data evidence based. Adopted by many facilities |
| Use of TBAs and CHWs in follow-up of mothers who don’t show up for their ANC clinic appointments | TBAs/CHWs are given names of ANC mothers who did not attend the previous ANC visit to follow them up.  Monthly meetings with TBAs/CHWs to share data  TBAs/CHWs are members of QIT and meet after every two weeks to give report. | Rated very high with a score of18/20  Active TBAs and CHWs are needed to make this idea work.  Availability of updated data in health facilities is also mandatory. |
| Having community dialogue meetings with CORPs and other community groups. | Identification of dates for CORPs/group meetings and use that as an opportunity.  Involvement of local leaders to organize for meetings.  Use of chiefs’ barazas.  Use of special forums like Education Through Listening sessions (a method of dialogue where participants educate each other through answering questions asked by the facilitator). | Scored16/20.  There are established active community groups which conduct dialogue meetings. |
| Making of a referral form to be used by TBAs in referring expectant mothers to the facility. | QIT decided to prepare forms to help track referrals from the community and also make it a form process of care. One of the QIT members drafted the letter which was reviewed and passed by the team. This draft letter was piloted with a few copies to selected TBAs and CHWs. When the idea was seen to work more copies were printed for all the available CHWs and TBAs. The facility management committee approved the funds for printing and photocopying. | Scored14/20, illiteracy among TBAs is the main challenge. |
| Formation of ANC support group which upon delivery the mother graduates to breastfeeding support group. In the group, the mothers meet regularly to discuss issues related to safe motherhood including having a birth plan. | ANC group existed before. The QIT team decided to strengthen this group. The group meets at the health facility and is spearheaded by the health worker. Clients were recruited on reporting for first ANC visit. They were given dates for ANC support group which occurs every fortnight. | About 6-10 mothers attend per visit. Most therefore get to attend a few visits before delivery.  Ranked lowly with a score of 8/20, because they are not easy to form and sustain. |
